# A Proposal for a Study on Treatment Selection and Lifestyle Recommendations in Chronic Inflammatory Diseases: A Danish Multidisciplinary Collaboration on Prognostic Factors and Personalised Medicine

**DOI:** 10.3390/nu9050499

**Published:** 2017-05-15

**Authors:** Vibeke Andersen, Uffe Holmskov, Signe Bek Sørensen, Mohamad Jawhara, Karina W. Andersen, Anette Bygum, Lone Hvid, Jakob Grauslund, Jimmi Wied, Henning Glerup, Ulrich Fredberg, Jan Alexander Villadsen, Søren Geill Kjær, Jan Fallingborg, Seyed A. G. R. Moghadd, Torben Knudsen, Jacob Brodersen, Jesper Frøjk, Jens F. Dahlerup, Ole Haagen Nielsen, Robin Christensen, Anders Bo Bojesen, Grith Lykke Sorensen, Steffen Thiel, Nils J. Færgeman, Ivan Brandslund, Allan Stensballe, Erik Berg Schmidt, Andre Franke, David Ellinghaus, Philip Rosenstiel, Jeroen Raes, Berit Heitmann, Mette Boye, Charlotte Lindgaard Nielsen, Lars Werner, Jens Kjeldsen, Torkell Ellingsen

**Affiliations:** 1 Focused Research Unit for Molecular Diagnostic and Clinical Research, IRS-Center Sonderjylland, Hospital of Southern Jutland, 6200 Aabenraa, Denmark; Signe.Bek.Sørensen@rsyd.dk (S.B.S.); Mohamad.Jawhara@rsyd.dk (M.J.); Karina.Winther.Andersen@rsyd.dk (K.W.A.); Anders.Bo.Bojesen@rsyd.dk (A.B.B.); Mette.Boye@rsyd.dk (M.B.); 2 Institute of Molecular Medicine, University of Southern Denmark, 5000 Odense, Denmark; uholmskov@health.sdu.dk (U.H.); GLSorensen@health.sdu.dk (G.L.S.); 3 OPEN, University of Southern Denmark, 5000 Odense, Denmark; 4 Institute of Regional Health Research, University of Southern Denmark, 5000 Odense, Denmark; Ivan.Brandslund@rsyd.dk; 5 Department of Dermatology and Allergy Centre, Odense University Hospital, 5000 Odense, Denmark; Anette.Bygum@rsyd.dk (A.B.); Lone.Hvid@rsyd.dk (L.H.); 6 Department of Clinical Research, University of Southern Denmark, 5000 Odense, Denmark; Jakob.Grauslund@rsyd.dk (J.G.); jwied@health.sdu.dk (J.W.); 7 Department of Ophthalmology, Odense University Hospital, 5000 Odense, Denmark; 8 Diagnostic Centre, Silkeborg Regional Hospital, University of Aarhus, 8600 Silkeborg, Denmark; Henning.Glerup@silkeborg.rm.dk (H.G.); ulrifred@rm.dk (U.F.); janvilla@rm.dk (J.A.V.); soekjaer@rm.dk (S.G.K.); 9 Department of Gastroenterology and Hepatology, Aalborg University Hospital, 9100 Aalborg, Denmark; jaf@rm.dk; 10 Department of Internal Medicine, Regional Hospital Herning, 7400 Herning, Denmark; seyeghan@rm.dk; 11 Department of Gastroenterology Hospital of South West Jutland, 6700 Esbjerg, Denmark; Torben.Knudsen@rsyd.dk (T.K.); Jacob.Broder.Brodersen@rsyd.dk (J.B.); Jesper.Frojk2@rsyd.dk (J.F.); 12 Department of Hepatology and Gastroenterology, Aarhus University Hospital, 8000 Aarhus, Denmark; Jensdahl@rm.dk; 13 Department of Gastroenterology D112, Herlev Hospital, University of Copenhagen, 2730 Herlev, Denmark; Ole.Haagen.Nielsen@regionh.dk; 14 Musculoskeletal Statistics Unit, The Parker Institute, Bispebjerg and Frederiksberg Hospital, 2000 Frederiksberg, Denmark; Robin.Christensen@regionh.dk; 15 Department of Biomedicine, Aarhus University, 8000 Aarhus, Denmark; ST@biomed.au.dk; 16 Department of Biochemistry and Molecular Biology, Villum Center for Bioanalytical Sciences, University of Southern Denmark, 5000 Odense, Denmark; Nils.F@bmb.sdu.dk; 17 Department of Clinical Biochemistry, Vejle Hospital, 7100 Vejle, Denmark; 18 Department of Health Science and Technology, Aalborg University, 9100 Aalborg, Denmark; as@hst.aau.dk; 19 Department of Cardiology, Aalborg University Hospital, 9100 Aalborg, Denmark; ebs@rn.dk; 20 Institute of Clinical Molecular Biology, Christian-Albrechts-University of Kiel, 24105 Kiel, Germany; a.franke@mucose.de (A.F.); d.ellinghaus@ikmb.uni-kiel.de (D.E.); p.rosenstiel@mucosa.de (P.R.); 21 Department of Microbiology and Immunology, Rega Institute, KU Leuven-University of Leuven, 3000 Leuven, Belgium; jeroen.raes@kuleuven.be; 22 VIB Center for Microbiology, 3000 Leuven, Belgium; 23 Department of Public Health, Section for General Medicine, University of Copenhagen, 1353 Copenhagen, Denmark; Berit.Lilienthal.Heitmann@regionh.dk; 24 Research Unit for Dietary Studies, The Parker Institute, Bispebjerg and Frederiksberg Hospital, Part of the Copenhagen University Hospital, 2000 Frederiksberg, Denmark; 25 The Danish Colitis-Crohn Association, 5000 Odense, Denmark; info@ccf.dk; 26 The Danish Psoriasis Association, 2630 Tåstrup, Denmark; lw@psoriasis.dk; 27 Department of Medical Gastroenterology, Odense University Hospital, 5000 Odense, Denmark; Jens.Kjeldsen@rsyd.dk; 28 Department of Rheumatology, Odense University Hospital, 5000 Odense, Denmark; Torkell.Ellingsen@rsyd.dk

**Keywords:** biomarker, exercise, food, molecular epidemiology, personalized medicine, patient related outcome measures (PROMs), red meat, smoking, treatment outcome, western style diet (WSD)

## Abstract

Chronic inflammatory diseases (CIDs), including Crohn’s disease and ulcerative colitis (inflammatory bowel diseases, IBD), rheumatoid arthritis, psoriasis, psoriatic arthritis, spondyloarthritides, hidradenitis suppurativa, and immune-mediated uveitis, are treated with biologics targeting the pro-inflammatory molecule tumour necrosis factor-α (TNF) (i.e., TNF inhibitors). Approximately one-third of the patients do not respond to the treatment. Genetics and lifestyle may affect the treatment results. The aims of this multidisciplinary collaboration are to identify (1) molecular signatures of prognostic value to help tailor treatment decisions to an individual likely to initiate TNF inhibitor therapy, followed by (2) lifestyle factors that support achievement of optimised treatment outcome. This report describes the establishment of a cohort that aims to obtain this information. Clinical data including lifestyle and treatment response and biological specimens (blood, faeces, urine, and, in IBD patients, intestinal biopsies) are sampled prior to and while on TNF inhibitor therapy. Both hypothesis-driven and data-driven analyses will be performed according to pre-specified protocols including pathway analyses resulting from candidate gene expression analyses and global approaches (e.g., metabolomics, metagenomics, proteomics). The final purpose is to improve the lives of patients suffering from CIDs, by providing tools facilitating treatment selection and dietary recommendations likely to improve the clinical outcome.

## 1. Introduction

Inflammatory bowel disease (IBD), encompassing Crohn’s Disease (CD) and ulcerative colitis (UC), is a member of a large family of diseases of the immune system that results in chronic inflammatory diseases (CIDs). Other CIDs are psoriasis (PsO), psoriatic arthritis (PsA), rheumatoid arthritis (RA), spondyloarthritides (ankylosing spondylitis, spondyloarthropathy, spondyloarthritis), spondylarthritis, AS), hidradenitis suppurativa (HS), and immune-mediated uveitis (UV).

CIDs have a large impact on individual patients and society. They are recurring, lifelong, potentially early onset illnesses that substantially affect the life quality of the patients and their families [1,2,3]. CIDs are complex diseases with both genetic and environmental factors involved in the disease development. Some genetic and environmental susceptibility factors are shared between the CIDs whereas other factors might differ [4,5,6,7,8,9,10,11,12,13,14,15,16,17,18,19,20,21,22,23,24,25]. CIDs are frequent; IBD affects up to 0.5% of the population in the Western world [26] and RA and PsO have global prevalences of 0.3–1.0% and 1.5%, respectively [27,28]. Furthermore, the disease burden is predicted to rise dramatically due to population growth, increasing age, and increasing disease incidence [29,30,31]. Therefore, in the future, a large and increasing challenge will be put on the health care system, as more patients will need treatment.

Personalised medicine may help to improve medical treatment and optimise the use of the health care resources. According to the EU: “Personalised Medicine refers to a medical model using characterisation of individuals’ phenotypes and genotypes (e.g., molecular profiling, medical imaging, lifestyle data) for tailoring the right therapeutic strategy for the right person at the right time, and/or to determine the predisposition to disease and/or to deliver timely and targeted prevention” [32]. In order to be able to choose the right therapeutic strategy for patients, tools that are able to predict treatment outcome are highly warranted. Drugs targeting the pro-inflammatory cytokine tumour necrosis factor-α (TNF), the so-called TNF inhibitors, are used for severe CIDs [33,34,35,36] and act through targeting and neutralising the effect of TNF, thereby diminishing the downstream effects of TNF. However, the pharmacodynamics of anti-TNF drugs seem to depend on other factors than simply the TNF-binding capacities [37]. Hence, their precise mechanism of action remains unclear. Nevertheless, although TNF inhibitors have proven to be highly effective for a majority of patients, a significant proportion of the patients do not respond to the treatment [38,39,40,41]. Thus genetics, environment in the form of lifestyle, and gene-environment interactions may affect treatment outcomes.

We therefore set out to identify molecular signatures able to predict response from anti-TNF therapy and to identify lifestyle factors that support achievement of optimal treatment outcomes. This study protocol describes the establishment of a cohort in order to obtain this information. The ultimate aim is to improve the lives of CID patients by providing tools for treatment selection and dietary recommendations for improved treatment outcome.

## 2. Defining Study Hypotheses to Be Evaluated

### 2.1. Biomarkers Predicting Response to Anti-TNF Drugs

Genetic architecture may define patient responses to anti-TNF drugs [42,43,44,45,46,47,48]. Patients may not respond to the treatment (i.e., “primary non-responders” due to, for example, genetics) or lose the effect over time (i.e., “secondary non-responders” due to, for example, development of anti-TNF antibodies) [37,49,50]. We have performed an exploratory study, where we found that a genetically determined strong TNF-mediated inflammatory response was associated with beneficial (primary) response to TNF inhibitor treatment, whereas genetically determined strong interleukin (IL)-1β, IL-6, and interferon-γ (IFN) responses were associated with no-response [43]. However, variants in genes encoding microbial-associated molecular pattern (MAMP) receptors such as Toll-like receptors (TLRs) and nucleotide oligomerization domain (NOD)-like receptors (NLRs) have been associated with high levels of TNF, IL-1β, and IL-6 in both humans and mouse IBD models [51]. These results suggest that different genetic variants may result in similar molecular profiles. This interpretation is in accordance with yet another study that found a significant increase in circulating levels of TNF, IL-1β, and IFN in IBD patients as compared to healthy donors [52]. Whether genetic variants (that hold the advantage of not changing over time) or, alternatively, molecular signatures (e.g., proteomics, metabolomics) may potentially stratify patients according to expected treatment results thus warrants further investigations. Therefore, one of the main hypotheses to be tested in the present study is whether specific genetic profiles (including TNF, IL-1β, IL-6 and IFN, and IL-10) may be identified among the CID patients that are associated with treatment outcome. IL-10 is included as it is associated with IBD [53,54] and has been found to downregulate TNF and IL-1β [55,56]. Additionally, the molecular mechanism that drives the inflammation in the individual patient will be sought identified.

### 2.2. Potential Lifestyle Factors That May Affect Treatment Response

We hypothesize that lifestyle factors, including diet, may impact treatment response to TNF inhibitor therapy. Human and animal studies suggest that diet and other lifestyle factors may affect the host immune system by various mechanisms that could potentially promote either healing or disease processes [57]. So far, very few high-quality prospective studies have evaluated the impact of lifestyle factors on anti-TNF treatment. More studies have been found focusing on diet and IBD, which intuitively connects with diet more than other CIDs. Therefore, the purpose of this section is to identify potential lifestyle factors for further investigations in relation to anti-TNF therapy. In order to explore different hypotheses, we have included studies that may be subject to recall bias, and bias introduced by lifestyle changes due to the disease itself. For review of evidence-based impact of diet on anti-TNF response, please refer to, for example, Richman and Rhodes [58] and Andersen et al. [59].

Lifestyle factors suggested affecting anti-TNF response:

● Smoking

In PsO, CD, and seropositive RA, smoking is associated with the risk of flaring disease and an aggressive disease course, whereas in UC, former smoking is associated with risk of disease and a severe course [60,61,62,63,64,65]. A gene-smoking interaction study found that smoking interacted with, for example, *NOD2* and *IL10*, and seven loci were found to interact differentially with CD and UC (Yadav et al., accepted [66]).

● Physical activity

High physical activity was found to be associated with low risk of relapse in CD and UC [67]. The potential effects of physical training are, however, not clear. Moreover, high body mass index may be involved in CIDs [68,69] and potentially in anti-TNF treatment. Macrophage accumulation in adipose tissue leads to release of inflammatory mediators that may enhance chronic inflammation [70]. The increased infiltration of the visceral adipose tissue by macrophages and adipocytokines leads to an inflammatory transformation of the visceral adipose tissue into creeping fat and potentially dysbiosis and intestinal translocation of bacteria [70].

● Dietary fibres

Dietary fibres from grains, fruit, and vegetables are metabolised by the gut microbiome to short chain fatty acids, including butyrate, propionate, and acetate, that represent important fuel for the intestinal mucosa and are associated with mainly anti-inflammatory effects. We recently proposed a mechanism whereby fibre intake may affect an anti-TNF response [71]. Low intake of dietary fibre may potentially change the gut microbial metabolism from using microbially derived short chain fatty acids to mucinous carbohydrates as the main energy source [72]. This might lead to degradation of the mucus layer [73]. The resultant hydrogen sulphide from mucus degradation may further reduce the disulphide bonds in the mucus network, rendering the mucus layer penetrable to bacteria and other compounds [73,74]. The result is an exposure of the intestinal epithelium to microbes and potentially other compounds. Next, molecular structures in microbes known as microbial- or pathogen-associated molecular patterns may activate pattern-recognition receptors such as TLRs, thereby activating the nuclear factor-ĸB (NFĸB) signalling pathway, resulting in inflammation [75].

A nested case-control study using the Swedish Västerbotten Intervention Program (VIP) cohort with a prospectively sampled dietary survey assessed dietary patterns among 386 individuals who developed RA and 1886 matched controls. An association was found between the highest tertile of carbohydrate-restricted diet and RA. However, the association was no longer statistically significant after adjustment for sodium intake [76].

A study on the Nurses’ Health Study (NHS) prospective cohort found that high fibre intake was associated with low risk of CD [13].

● Meat, protein, fat, and dietary sulphur

Red and processed meat is a rich source of protein, fat, and dietary sulphur that in various ways may promote inflammation. For example, colonic bacterial fermentation may lead to the formation of pro-inflammatory branched chain fatty acids, ammonia (NH3), and hydrogen sulphide (H2S) [77,78]. Hydrogen sulphide resulting from high intake of meat and other sulphur-containing compounds may reduce the disulphide bonds in the mucus network, rendering the mucus layer penetrable to bacteria and other compounds [73,74]. Eventually, microbes may reach the epithelium and activate TLRs and next the NFĸB inflammatory pathway, as described above.

High intake of red meat and total protein has in a prospective cohort been associated with a risk of developing inflammatory polyarthritis [25].

Another study suggested that high consumption of meat, particularly red and processed meat, protein, and alcohol may increase the risk of relapse in patients with UC, and that high sulphur or sulphate intake may offer an explanation for the observed increased likelihood of relapse [79].

On the other hand, high protein intake is recommended in some patients with active IBD (1.2–1.5 g/kg/day in adults) relative to that recommended for the general population [80].

● *n*-3 and *n*-6 polyunsaturated fatty acids (PUFAs)

The essential *n*-6 PUFA linoleic acid is present in red meat (particularly beef and pork), various cooking oils, and certain margarines. Linoleic acid undergoes metabolic conversion to arachidonic acid. Arachidonic acid incorporated in the cell membrane may if released be metabolised to pro- and anti-inflammatory eicosanoids (i.e., prostaglandins and leukotrienes). A study using the European Prospective Investigation into Cancer and Nutrition (EPIC) cohort found that the highest quartile of intake of linoleic acid was associated with an increased risk of UC (odds ratio (OR) = 2.49, 95% confidence interval (CI) = 1.23 to 5.07, *p* = 0.01), whereas the highest quartile of intake of the *n*-3 PUFA docosahexaenoic acid was associated with a reduced risk of UC with an odds ratio of 0.23 (95% CI = 0.06 to 0.97) [81]. Another study of the same cohort found that the highest quintile of intake of *n*-3 PUFA was associated with a reduced risk of CD (OR = 0.07; 95% CI = 0.02–0.81) [16].

● Vitamins and carotenoids

Vitamin D deficiency has been assessed in the NHS cohort and was found to be associated with risk and severe disease course of CD [11,82], but not of RA [83].

A systematic review of prospective studies that examined dietary intake prior to the onset of RA found that high consumption of olive oil, oil-rich fish, fruit, vegetables, and the carotenoid beta-cryptoxanthin, a vitamin A precursor found in citrus, was suggested to protect against RA development [84]. Another study of 56 UC patients in remission compared symptoms (abdominal pain, faecal blood, mucus, and pus) with those recalled before diagnosis, and actual diet. A lack of abdominal pain, faecal blood, faecal mucus, and faecal pus during remission or no increase in frequency or intensity of symptoms compared to those observed before the UC diagnosis was interpreted as a lack of the above-mentioned symptoms. The authors found that retinoid intake was associated with changes of the symptoms [85].

● Sugar-sweetened soda

Finally, regular consumption of sugar-sweetened soda, but not diet soda, was found to be associated with increased risk of seropositive RA in women, independent of other dietary and lifestyle factors, in the prospective NHS study [86]. Another study suggested that the content of sulphite in soft drinks may be causally involved in worsening UC [87].

### 2.3. Gene-Environment Interactions Predicting Response to Anti-TNF Drugs

Emerging data suggests that diet may affect metabolic functions in the gut in a way that may out-compete genetically based differences. In a *Fut2*-deficient mouse model, differences in faecal metabolites were found between *Fut2*-deficient and wild type mice on a control diet, a difference that was lost while on a polysaccharide-deficient diet [88]. *FUT2* encodes fucosyltransferase-2 (FUT2), which mediates the inclusion of fucose in sugar moieties of glycoproteins, including those that are part of the intestinal mucus protecting the intestinal mucosa. Given that about 20% of the population has inactive FUT2 [89], that *FUT2* is associated with CD [89,90], and that products from mucus degradation have been found to affect intestinal mucosal function [91], this example shows the potential importance of diet and gene-diet interactions on treatment response.

## 3. Aims and Hypotheses

The purposes of the present study are twofold: (1) to identify molecular profiles of prognostic value to help tailor treatment decisions to an individual or group of individuals with CID initiating anti-TNF therapy (stratified medicine research); and (2) to identify a lifestyle that may support the achievement of an optimal treatment response to TNF inhibitors.

## 4. Materials and Methods

### 4.1. Study Design

A prospective cohort study of anti-TNF naïve patients that initiate their first TNF inhibitor treatment will be established. The patients will be investigated two times, before initiating anti-TNF therapy and while on this therapy (T = 2). The endpoint is the treatment outcome defined as A: Responder (drug-continuation) or B: Non-responder (B1: Drug-discontinuation due to lack of effect or B2: Unacceptable side-effects). This evaluation will be based on disease activities, using clinical scores and laboratory data, and shared decision making between patient and physician using standardized guideline approved patient reported outcomes (PROMs) according to national guidelines for each CID recommended in the respective national guidelines [92]. The enrolment period will start in 2017 and run for 2 years.

### 4.2. Participants

In total, more than 300 CID patients will be enrolled including 45 CD patients and 55 UC patients. Patients will be investigated before initiation of and on TNF inhibitors. Patients will be reinvestigated 14–16 weeks after initiation.

### 4.3. Clinical Data Sampling

Clinical data will consist of personal data (e.g., gender, age, weight, body mass index), health data (e.g., diagnosis/diagnoses, year of diagnosis, medication, and comorbidity), disease activity (disease activity scores, laboratory data, shared decision making between patient and physician using PROMs), and lifestyle data (e.g., diet, smoking, alcohol consumption, physical activity). The first and the second investigation will be similar except for the sampling of diet information on the second visit, where only changes since the first visit will be registered.

Clinical data will be collected using a questionnaire and registries. Registry data will be retrieved from the unique Danish registries using the Danish individual civil registration number (CPR) including BIO-IBD [93], DANBIO [94], DERMBIO [95] (database on IBD, RA, HS, AS, PsA, and PsO patients on biological therapy), the National Patient Registry (e.g., comorbidity), registries on medication and use of receipts, local laboratory databases, and the electronic patient records. In addition, The Danish Biobank and Patobanken will be used for retrieval of potential additional biological samples. Clinical data (e.g., body weight, height, results of routine blood samples) will be sampled. Furthermore, each participant will fill out a questionnaire, thereby providing information on disease activity, quality of life, and lifestyle including diet. Disease activities and quality of life will be registered by validated questionnaires (e.g., presence of abdominal pain, faecal blood, and altered bowel habit [96], Mayo Clinic Score, Simple Clinical Colitis Activity Index (SCCAI), Harvey-Bradshaw index (HBI), Health Assessment Questionnaire 1 (HAQ1), Short Health Scale (SHS), American College of Rheumatology criteria (ARC 20/50/70), Psoriasis Area and Severity Index (PASI 75), HiSCR, uveitis treatment failure, and Standardization of Uveitis Nomenclature for Reporting Clinical Data (SUN)). Lifestyle will be registered using a validated food-frequency questionnaire (FFQ) that includes portion size and questions on smoking, physical activity, alcohol consumption, and use of over-the-counter medicine (anti-diarrhoea agents and painkillers) [97,98]. The questionnaire is in Danish language. All data will be stored in a secure research storage facility [99].

### 4.4. Biological Specimen Sampling

From all participants, blood, urine, and faeces will be sampled. In addition, IBD intestinal biopsies will be sampled (Table 1 and Table 2). In selected cases, additional biological material on participants from this study may be retrieved from Patobanken and the Danish Biobank.

### 4.5. Biological Analyses

- Molecular analyses (genetics, transcriptomics, proteomics, metabolomics, gut microbiota).
- Fluorescent in situ hybridization (FISH) on formalin-fixed and paraffin-embedded intestinal tissue.
- Fatty acid distribution in erythrocytes.
- Plasma biomarkers for inflammatory and tissue degradation processes.
- Routine blood analyses (e.g., haemoglobin, leucocytes, HbA1c, cholesterol and lipoprotein characterisation, C-reactive protein (CRP)).
- Advanced blood analyses (e.g., cytokines, interleukins, complement split products, sugar and leucocyte activation markers, protein glycosylation).

### 4.6. Data Analyses

We will use this rigorously designed, prospective cohort study to explore our ability to predict clinical response across the conditions included, and explore whether diet is an informative prognostic factor. Per default, the statistical model will include condition (any of the CID conditions included) and clinical centre as fixed effects. Functional annotation of the identified single nucleotide polymorphisms SNPs, gene prioritization, and pathway and tissue/cell type enrichment analyses will be performed using publicly accessible databases (e.g., SIFT [100], DANN [101], HaploReg [102], GTEx [103], DEPICT [104]). Development of a shared platform for data integration using advanced bioinformatics tools is highly prioritized. Data will be integrated in a descriptive model of molecular signatures for predicting treatment results and lifestyles supporting beneficial treatment results for the individual patient. This model will be used for further exploitation using bioinformatics tools.

- We will identify lifestyle factors alone or combined (e.g., high meat and low fibre), lifestyle-associated biomarkers, and molecular signatures associated with treatment response among anti-TNF treated CID patients.
- We will evaluate shared and unique molecular signatures associated with treatment response between the individual CIDs (e.g., gut microbial composition [105,106]).
- We will evaluate the mechanisms underlying specific dietary items by analysing their pathway from intake to being metabolized. For example, for high meat intake we will evaluate the impact on faeces microbes and metabolism, mucosal metabolism (in IBD patients) and immune response, and urine metabolites.
- In addition, we will evaluate shared and unique molecular signatures associated with one or more CIDs.
- We will validate surrogate markers (lifestyle-associated biomarkers) of lifestyle factors in a control group of patients referred for endoscopic investigation that had a normal endoscopic examination. These patients will be investigated once and undergo the same examinations as the IBD patients at visit 1.
- We will explore potential gene-environment interactions and their biological mechanisms in both CID and the control group (e.g., intake of red and processed meat/cereal/fibre/fruit/vegetables/legumes/use of alcohol and *IL10*, *FUT2*, *ABCB1*, *PTGS2*, *NFKB1*, *IL1B*, and *TLRs*).

*Sample size considerations*: It is a well-known difficulty for exploratory prognostic factor research studies like this to formalize how many participants (i.e., with events) to include. In order to consider an adequate number of outcome events, we are applying “the rule of thumb” that dictates that 10 outcome events are needed for each independent variable (possible predictors). We plan to enrol 320 patients in total, and anticipate that 50% of these will experience a clinical response during the 14–16 week period after therapy with TNF inhibitors is initiated. With this in mind, in anticipating that we will see at least 160 clinical responses (among the 320 patients), we will have a reasonable power to explore the impact of as many as 16 independent (predictor) variables (including condition and clinical centre). If we focus on the contrast between groups, for a comparison of two independent binomial proportions using Pearson’s Chi-square statistic with a Chi-square approximation with a two-sided significance level of 0.05 (*p* < 0.05), a total sample size of 318 assuming an allocation ratio of 1 to 2 has an approximate power of 0.924 (i.e., >90% statistical power) when the proportions responding are 60% and 40%, respectively).

### 4.7. Evaluation of Results with Potential Interest for Patient Care or the Public in Other Cohorts

Efforts will be made to replicate interesting findings with potential prognostic value in other cohorts of anti-TNF treated patients (via the International IBD Genetic Consortium (IIBDGC) and other consortia, including German and Spanish cohorts of anti-TNF treated IBD and RA patients) and in other cohorts of CID patients (e.g., gene-environment analyses of CID cases from the Danish “Diet, Health and Cancer” cohort and potentially other cohorts with lifestyle data [107,108]).

### 4.8. Organisation and Patient Research Partners (PRPs)

We have chosen a multidisciplinary approach, ensuring that the highest competences are present for study planning and accomplishment. The project has been organised with a Clinical Research Group and an Analytical Research Group. The clinical group includes representatives from the medical, gastroenterological, rheumatological, dermatological, and ophthalmological departments that are sampling the cohort. The analytical group will perform the analyses on the biological material. Furthermore, the project has been organised with a steering committee including the project initiator (Principal Investigator (PI)). The steering committee is responsible for the scientific follow-up and will be organising meetings for the involved parties. The PI has planned and organised the study and is responsible for legal permissions. The whole group including clinicians and analysts is responsible for the scientific results and the budget.

Collaboration between patients and health care professionals on research projects is relatively new [109,110,111]. Involvement of patients in research (patients research partners (PRPs)) should result in patients’ views, for example, on prioritising being heard and incorporated. Furthermore, individual patients and patient organisations may help in designing research studies, preparing information material, discussing results, dissemination of results, and recruitment of study participants. Recommendations include relevant support to and education of PRPs. With this initiative, we want to get experiences with using PRPs. This project includes the Danish Colitis-Crohn Association, which is represented by director Charlotte Lindgaard Nielsen, the Danish Psoriasis Association represented by director Lars Werner, and two individual RA patients from one of the involved departments.

### 4.9. Dissemination of Results to the Public and Scientifically

Target journals include nutritional journals and specific journals for immunology, gastroenterology, rheumatology, dermatology, and ophthalmology. In addition to the scientific reporting of results, major findings with translational implications will be communicated to health care professionals, patient organisations, public health policy makers, and to the general public through various media and news activities.

### 4.10. Ethics

Written informed consent will be obtained from all participants before participation in the study. The project has been approved by The Regional Scientific Ethical Committee (S-20160124) and the Danish Data Protection Agency (2008-58-035).

## 5. Discussion

This project overview describes a series of studies that will reveal prognostic factors of interest for the management of patients with CIDs. Thus, the establishment of a prospective cohort will hopefully help improve the lives of CID patients by providing dietary recommendations that support optimal outcomes of anti-TNF treatment and tools for selecting the right treatment for the right patient.

Identifying the molecular mechanism that drives the inflammation in the individual patient may represent a therapeutic target [43]. The reason that drugs targeting, for example, IL-1β have not shown efficiency in clinical trials could be due to the fact that the drug was tested on unselected groups of patients. Yet, the drug may have an effect on a subgroup of patients selected on the basis of their individual genetic profile. In accordance, blockades of IL-1β (Anakinra) and IL-6 (Tocilizumab) have shown efficiency in some patients with RA, PsO, and UV [112,113,114,115,116].

Among the advantages of the study is the prospective design, i.e., the treatment results are not known at the time of the sampling of lifestyle factors, and the well-characterised study participants, both clinically and biologically. The Danish registries, including databases on the various anti-TNF treated CIDs, the National Patient Registry, local databases, and electronic patient records, are to be used to retrieve data for characterisation of the patients.

One disadvantage of the study is the large questionnaire of lifestyle, quality of life, and disease activity that has to be answered by the participants. In addition, relatively low numbers of participants will be collected. Therefore, in order to increase power, we will collaborate with other similar projects going on in, for example, IIBDGC and other large consortia. In fact, we are currently performing genome-wide genotyping of more than 2500 Danish CID patients with response data to anti-TNF therapy. Thereby, we will identify new candidate genes for defining the outcome of TNF inhibitors. These candidate genes can be further evaluated in the current cohort including genotype-phenotype characterisation. Additionally, in the near future, other cohorts for studying impact of diet on treatment response will emerge and offer collaborative possibilities.

As this study is an explorative study and because of the number of tests, most of the obtained results should be replicated in other cohorts before their validity can be assessed.

## 6. Conclusions

According to the Strategic Research and Innovation Agenda (SRIA), European Union, “Personalised Medicine represents one of the most innovative new concepts in health care. It holds real promise for more effective early diagnosis and more effective and less toxic treatments for patients, for improved medical service to citizens, and for improving the overall health of the population” [32]. CIDs are some of the most challenging medical conditions; they affect a huge number of persons, in many cases through large parts of their lives. Molecular profiles may identify the driving mechanisms in the diseased individuals and may serve as guidance for treatment selection and for lifestyle recommendations that may eventually improve the lives of the patients.

## Figures and Tables

**Table 1:** Participant samples available for the study.

Participants	Clinical Data	Blood	Faeces	Urine	Intestine
*Before initiation of anti-TNF treatment*					
IBD	100	100	100	100	100
RA/others	220	220	220	220	
*On anti-TNF treatment*					
IBD	100	100	100	100	100
RA/others	220	220	220	220	

Abbreviations: IBD, inflammatory bowel disease; RA, rheumatoid arthritis; others: psoriasis, psoriatic arthritis, spondyloarthritides (ankylosing spondylitis, spondyloarthropathy, spondyloarthritis, spondylarthritis), hidradenitis suppurativa, immune-mediated uveitis; anti-TNF, drugs targeting the pro-inflammatory cytokine tumour necrosis factor-α.

**Table 2:** Overview of biological samples available for this study.

Biologic Material		Participants		Visits		
	**Method**	**Particip**	**N Particip**	**Non-IBD**	**IBD**	**Total**
Blood	EDTA	all	320	440	200	640
Blood	empty	all	320	440	200	640
Blood	heparin	all	320	440	200	640
Blood	Na-citrate	all	320	440	200	640
Blood	PAXgene	all	320	440	200	640
Urine	empty	all	320	440	200	640
Faeces	empty	all	320	440	200	640
Faeces	RNAlater	all	320	440	200	640
Biopsies	FFPE	IBD	100	-	200	200
Biopsies	RNAlater	IBD	100	-	200	200
Biopsies	N2	IBD	100	-	200	200

Samples are processed according to standard operating procedures (SOPs). Abbreviations: EDTA; ethylenediaminoetetraacetic-acid, Particip; Participants, FFPE; formalin-fixed and paraffin embedded.
